# Screening for Core Genes Related to Pathogenesis of Alzheimer’s Disease

**DOI:** 10.3389/fcell.2021.668738

**Published:** 2021-04-22

**Authors:** Longxiu Yang, Yuan Qin, Chongdong Jian

**Affiliations:** ^1^Department of Neurology, The First Affiliated Hospital of Guangxi Medical University, Nanning, China; ^2^Department of Neurology, The Affiliated Hospital of Youjiang Medical University for Nationalities, Baise, China

**Keywords:** Alzheimer’s disease, IFS, feature selection, KNN model, protein-protein interaction network

## Abstract

Alzheimer’s disease (AD), a nervous system disease, lacks effective therapies at present. RNA expression is the basic way to regulate life activities, and identifying related characteristics in AD patients may aid the exploration of AD pathogenesis and treatment. This study developed a classifier that could accurately classify AD patients and healthy people, and then obtained 3 core genes that may be related to the pathogenesis of AD. To this end, RNA expression data of the middle temporal gyrus of AD patients were firstly downloaded from GEO database, and the data were then normalized using *limma* package following a supplementation of missing data by k-Nearest Neighbor (KNN) algorithm. Afterwards, the top 500 genes of the most feature importance were obtained through Max-Relevance and Min-Redundancy (mRMR) analysis, and based on these genes, a series of AD classifiers were constructed through Support Vector Machine (SVM), Random Forest (RF), and KNN algorithms. Then, the KNN classifier with the highest Matthews correlation coefficient (MCC) value composed of 14 genes in incremental feature selection (IFS) analysis was identified as the best AD classifier. As analyzed, the 14 genes played a pivotal role in determination of AD and may be core genes associated with the pathogenesis of AD. Finally, protein-protein interaction (PPI) network and Random Walk with Restart (RWR) analysis were applied to obtain core gene-associated genes, and key pathways related to AD were further analyzed. Overall, this study contributed to a deeper understanding of AD pathogenesis and provided theoretical guidance for related research and experiments.

## Introduction

Alzheimer’s disease (AD) is a progressive neurodegenerative disorder that is almost incurable. According to the World Alzheimer Report 2018, there were approximately 50 million patients worldwide who suffered from AD, and AD became a major cause of death among old people (Patterson, 2018). Its main features are the deposit of β-amyloid (Aβ) plaques and neurofibrillary agglomerates (Dos Santos Picanco et al., 2018). A recent genetic study unearthed that Aβ deposition frequently occurs in people with ApoE4 (Genin et al., 2011). People with ApoE4 gene have high plasma cholesterol, which in turn stimulates the deposition of Aβ and tau proteins in the brain, thereby leading to AD (Greenberg et al., 2020). It is reported that the pathogenesis of AD is associated with heredity and gene expression like TREM2, PLCG2, ABI3 (Sims et al., 2017; Ulland and Colonna, 2018). Besides, circRNA and miRNA are also found to be related to the pathogenesis of AD (Dube et al., 2019; Iranifar et al., 2019).

Gene expression regulation is the most critical way of life regulating. Aberrant gene expression in brain tissue accounts for diverse diseases. For instance, ROCK1 gene expression is relevant to AD progression (Li X. et al., 2020). While aberrant expression of genes such as LRRK induces the occurrence of Parkinson’s disease (Wang et al., 2017). Researchers disclosed that gene expression in the MTG is probably closely related to the pathogenesis of AD, and the blockage of GABA signaling pathway in the MTG may result in cognitive decline (Govindpani et al., 2020). Given the above studies, this study surmised that the gene expression of the MTG is closely related to the pathogenesis of AD. This study attempted to probe into the critical factors affecting the pathogenesis of AD by analyzing the gene expression related to the MTG of AD sufferers.

Machine learning is a pivotal means of modern medical research, by which researchers always explore core genes that affect the occurrence of diseases. In the field of bioinformatics, machine learning is mainly applied in construction of diagnostic or prognostic models for disease, screening for biomarkers indicating disease outcome, etc., while linear-regression analysis is the common one in prognostic model establishment. For example, Feng and Jin (2018) constructed a risk model for prognostic prediction of patients with breast cancer through bioinformatics methods. Additionally, in algorithms that help for model construction, Support Vector Machine (SVM), Random Forest (RF), Artificial Neural Network (ANN), and K-Nearest Neighbor (KNN) are frequently used (Zhang, 2016; Huang et al., 2018; Chowdhury et al., 2019; Kulkarni et al., 2021). A previous study combined the KNN with genetic algorithm to greatly improve the accuracy of heart disease diagnosis (Jabbar et al., 2013). Max-Relevance and Min-Redundancy (mRMR) is an effective analytical method used to identify core genes in diseases. For example, Xu et al. (2014) identified core genes with mRMR to establish a model for determining malignant thyroid epithelioma. Regarding the identification of feature genes, the genes that are screened out to establish prognostic models or classifiers for disease are recognized potential biomarkers for outcome prediction. Besides, methods like mRMR, Boruta and ReliefF are also practicable (Zhang et al., 2008; Degenhardt et al., 2019). The mRMR method is instrumental for discovering core genes that affect Guillain-Barré syndrome (GBS) (Xu et al., 2016). This study selected the best AD classifier among SVM, RF, and KNN classifiers following the mRMR analysis and incremental feature selection (IFS) algorithm. Afterwards, functions of related genes in the optimal classifier were further explored. These findings may provide a deeper insight into the research and treatment of AD.

## Analytical Methods

### Dataset Preparing

**FIGURE 1:**
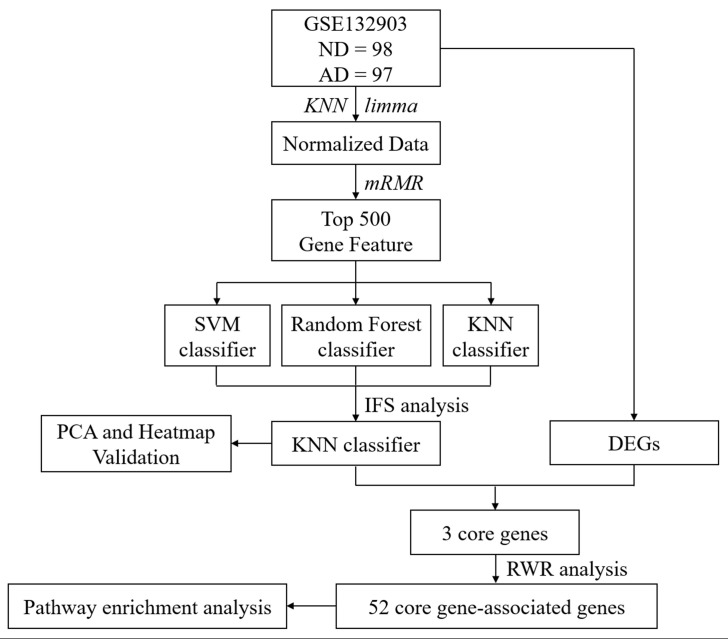
Overall workflow of this study.

As presented in Figure 1, the overall workflow of this study was drawn to clarify our research design. RNA expression data (GSE132903) of the AD MTG were downloaded from the Gene Expression Omnibus (GEO) database^1^. The samples in this dataset were collected from Brain and Body Donation Program (BBDP) volunteers, including 98 healthy subjects (ND) and 97 AD patients. The corresponding platform annotation file was downloaded to annotate the RNA expression dataset, and an expression matrix with gene ids was created with the probe annotation categories. Afterwards, the missing data were supplemented with KNN (*K* = 10) (Troyanskaya et al., 2001; de Brevern et al., 2004), and the final data were standardized using the R package *limma* for further analysis.

### Feature Selection by mRMR

The mRMR algorithm was implemented to rank feature importance in standardized cohort as previously reported (Xu et al., 2014, 2016; He et al., 2019). The mRMR feature selection method can compute relevance between features and sample phenotypes, and can comprehensively rank features according to the redundancy between features. Features at the top have a better trade-off between the relevance and redundancy than features at the bottom. Here, feature genes with the maximum relevance with AD and the minimum redundancy with other features were found through the mRMR algorithm (Peng et al., 2005). Mutual information (MI) represents the relevance between a random variable and another random variable. MI function was applied to assess the relevance between features and quantify the relevance and redundancy. The MI function was defined as follows (1),

(1) $$I\,\left(x,y\right)=\iint p\,\left(x,y\right)\,l\,o\,g\,\frac{p\,\left(x,y\right)}{p\,\left(x\right)\,p\,\left(y\right)}\,dx\,dy$$

where *x* and *y* represent two vectors, *p (x, y)* represents joint probabilistic density, *p(x)* and *p(y)* represent marginal probabilistic densities. Thereafter, the relevance between genes and sample phenotypes were computed following the MI function (2),

(2) D=I⁢(f,c)

where *D* represents the relevance between genes and phenotypes, *f* represents gene, and *c* represents phenotype. The redundancy between genes was identified as *R* and was computed by the following formula (3),

(3) $$R=\frac{1}{m}\,\sum_{f_{i}\in T}I\,\left(f,f_{i}\right)$$

where *m* represents the total number of genes in the dataset, and *T* represents the gene set containing all genes. Then, the trade-off between the relevance and redundancy was computed by the following formula (4),

(4) $$m\,a\,x_{f}\,\left(D-R\right)$$

After repeated computation per the above formula, the trade-off of each feature gene in the dataset was sorted. A new gene list was obtained (5),

(5) $$S=\left\{f_{1}^{\prime },f_{2}^{\prime },\ldots ,f_{h}^{\prime },\ldots ,f_{N}^{\prime }\right\}$$

where the subscript index of each feature gene in S was selected. A feature that was selected earlier had a smaller index and could have high feature importance. Finally, the top 500 genes in the ranked feature list were selected for subsequent research.

### Classifier Selection by IFS Method

Following mRMR analysis, IFS was sequentially applied to identify genes for the optimal AD classifier (Chen et al., 2017; Li M. et al., 2020). Firstly, based on the ranked feature list, a series of feature subsets were set as F_1_, F_2_, F_3_…F_*n*_, where F_*i*_ = {f_1_, f_2_…f_*i*_} and f_*i*_ refers to the top 500 genes in the ranked feature list. Secondly, the Python package *sklearn* was applied to establish a series of AD classifiers using the above feature subsets with SVM, RF, and KNN algorithms. SVM, RF, and KNN classifiers all can compute the expression of feature genes to identify AD patients (Zhang, 2016; Sarica et al., 2017; Huang et al., 2018). IFS curves were then plotted under 10-fold cross-validation to obtain the Matthews correlation coefficient (MCC), a parameter able to reflect classifier effectiveness (Chicco and Jurman, 2020), of each candidate classifier. Eventually, the classifier with the greatest MCC value was identified as the optimal AD classifier, and the genes involved in were taken as the optimal feature genes.

### Principal Component Analysis (PCA) and Heatmap Construction

The R package *FactoMineR* was applied for dimensionality reduction of the two downloaded cohort based on the features of the optimal classifier following PCA. In brief, PCA can reduce the dimensionality of the data in two datasets and map the data into 2 representative dimensions. A scatter plot was drawn based on the distribution of samples in the two dimensions to present the variance between samples and between groups. Furthermore, the expression of feature genes of the classifier in ND and AD populations was compared through clustering analysis using the R package *pheatmap*.

### Random Walk With Restart (RWR) and Enrichment Analyses

To explore core genes from the optimal classifier and their potential functions, R package *limma* was firstly employed to analyze the difference in gene expression between the ND and AD groups in GEO, and differentially expressed genes (DEGs) were screened (| Log2FC| > 0.585, FDR < 0.05). The DEGs were then intersected with the feature genes in the identified classifier to obtain core genes. Wilcox test was implemented to test the differential expression of the core genes in ND and AD samples. Additionally, the DEGs were projected onto the STRING website to construct a protein-protein interaction (PPI) network (Interaction Score > 0.4). RWR algorithm stimulates a random walking starting from a seed node or several seed nodes to a randomly selected neighbor node or to return to the origin in a constructed network. This walking is iterative and terminates when all nodes in the network are walked, and finally a relevance score between each node and the seed node is obtained (Kohler et al., 2008; Zhang et al., 2018). Here, RWR algorithm was run to calculate the relevance score between each node gene and the seed node gene, and the node gene with a score > 10^–5^ was taken as the core gene-associated gene.

Gene Ontology (GO) function annotation and Kyoto Encyclopedia of Genes and Genomes (KEGG) pathway enrichment analyses were sequentially carried out for the core gene-associated genes using the R package *ClusterProfiler*, thereby to explore the critical functions that may affect the pathogenesis of AD. The results were finally visualized using the R package *enrichplot*.

## Results

### Results of the mRMR and IFS Analyses

**FIGURE 2:**
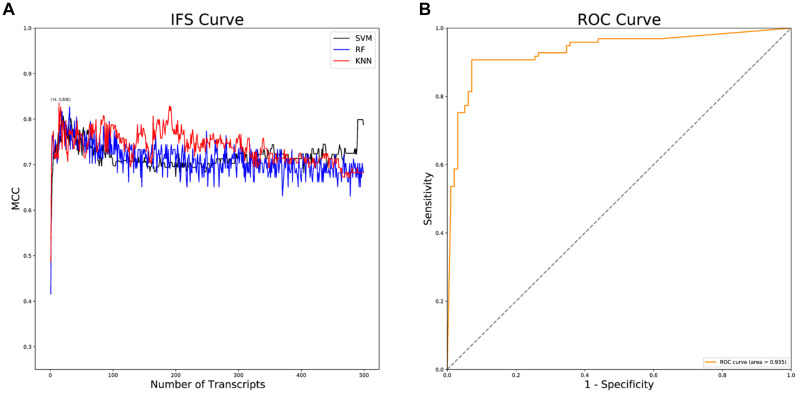
IFS and ROC analyses. **(A)** IFS curves of SVM, RF and KNN classifiers. The black curves indicate SVM classifiers; The blue curves indicate RF classifiers; The red curves indicate KNN classifiers; **(B)** ROC curve of the KNN classifier.

Following data downloading and normalization, 28,844 genes were obtained for feature importance analysis through mRMR analysis. The top 500 feature genes in the mRMR analysis were selected (Supplementary Table 1), by which a series of AD classifiers were constructed. Then, IFS analysis was implemented to select the optimal classifier. As illustrated in Figure 2A, the KNN classifier composed of 14 feature genes had the highest Matthews correlation coefficient (MCC) value. Then, the diagnostic efficacy of the KNN classifier was validated using receiver operation characteristic (ROC) curves. The results presented the sensitivity was 0.907, the specificity was 0.929, the accuracy was 0.918, the MCC value was 0.836, and the area under the curve (AUC) value was 0.935, indicating the high diagnostic efficacy of the KNN classifier in classifying AD patients accurately (Figure 2B).

### Results of PCA and Heatmap Analysis

**FIGURE 3:**
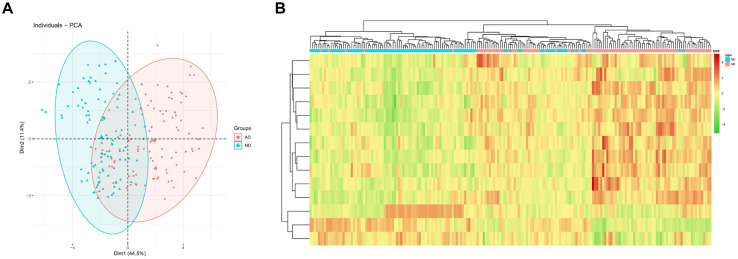
PCA and heatmap analysis based on the feature genes in the KNN classifier. **(A)** PCA showed diagnostic efficiency of the KNN classifier in ND and AD populations; **(B)** Heatmap showed expression of feature genes in the KNN classifier in ND and AD populations. The red means high expression while the green means low expression.

Principal Component analysis was conducted for two groups of patients (ND/AD) according to the expression of 14 feature genes in the optimal KNN classifier. The results revealed that PCA analysis could markedly classify AD patients and normal subjects (Figure 3A). Besides, a clustering heat map was drawn to analyze the expression of the 14 feature genes in different populations. The results denoted that the 14 feature genes in the KNN model could distinguish AD patients from healthy subjects (Figure 3B). These findings manifested that the 14 feature genes in the KNN model exhibited a favorable performance in classifying AD patients from normal individuals, indicating an outstanding diagnostic efficacy.

### Results of RWR and Enrichment Analysis

**FIGURE 4:**
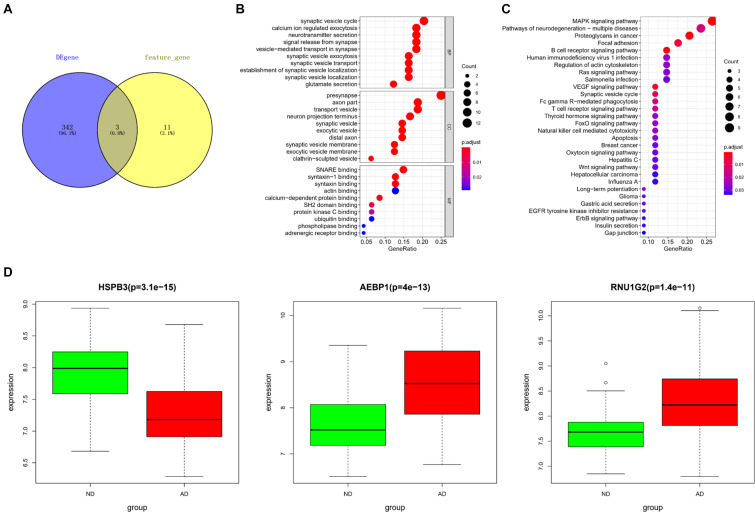
Core gene selection and functional enrichment analysis. **(A)** Venn diagram was drawn to select core genes between DEGs and feature genes in the KNN classifier; **(B,C)** Results of GO and KEGG enrichment analyses. The dot size means the number of genes enriched in corresponding terms; The dot color represents the significance of corresponding terms; **(D)** Expression of core genes (HSPB3, AEBP1, RNU1G2) in ND (green) and AD (red) populations.

Differentially expressed genes screened from the downloaded gene expression data in GEO and feature genes in the KNN classifier were intersected to obtain 3 core genes, including heat shock protein family B (small) member 3 (HSPB3), adipocyte enhancer binding protein 1 (AEBP1), RNA U1 Small Nuclear 4 (RNU1G2) (Figure 4A). Based on the DEGs, a PPI network was constructed. Since the Interaction Score of RNU1G2 in the PPI network was less than 0.4, AEBP1 and HSPB3 were picked up as seed nodes to perform RWR algorithm. Eventually, 52 core gene-associated genes were obtained (Supplementary Table 2). Then, the 52 core gene-associated genes were subjected to enrichment analysis. As analyzed, the genes were related to biological processes, such as synaptic vesicle cycle, transport vesicle, protein kinase C binding (Figure 4B), and activated in pathways such as MAPK signaling pathway, B cell receptor signaling pathway, and T cell receptor signaling pathway (Figure 4C). Finally, expression of the 3 core genes in AD was detected. As shown in Figure 4D, HSPB3 was conspicuously down-regulated, while AEBP1 and RNU1G2 were notably up-regulated in the AD group. Taken together, these results demonstrated that the 3 core genes were closely associated with the pathogenesis of AD, and were mainly related to cell functions involved in immunity and cell transportation.

## Discussion

The cause of AD is thought to be correlated with Aβ deposition and the hyperphosphorylation of tau proteins, whereas the cause of the above processes remains a mystery, which puzzles researchers for treatment and prevention of AD (Park et al., 2019; Busche and Hyman, 2020). There are many hypotheses about AD, including Aβ cascade hypothesis, tau hypothesis, inflammation hypothesis, cholinergic and oxidative stress hypothesis and glucose hypometabolism (Du et al., 2018). Aβ hypothesis considered as a major cause of AD believes that Aβ deposition is the major cause of AD, and Aβ deposition in the nervous system makes nerve cells lack necessary nutrients and cell apoptosis (Du et al., 2018). Neurofibrillary tangles are another pathological feature of AD patients, besides, phosphorylated tau aggregates proteins to cause neuron damage, and drugs targeting tau are promising for AD therapy (Brier et al., 2016; Gauthier et al., 2016; Li and Gotz, 2017; Novak et al., 2017). In addition, several investigations considered that inflammation and oxidative stress are central to AD pathogenesis. The sustained activation of the brain’s resident macrophages (microglia) exacerbates both Aβ and tau pathology and hastens AD pathogenesis (Kinney et al., 2018). Besides, the Aβ clearance disorder caused by oxidative stress also hastens AD pathogenesis (Cheignon et al., 2018). The deepening of study on the pathogenesis of AD has revealed numerous key genes affecting the pathogenesis of AD. For instance, MS4A and somatic APP are involved in the above pathways to influence AD pathogenesis (Lee et al., 2018; Deming et al., 2019). Moreover, lncRNA and miRNA also participate in AD pathogenesis (Dube et al., 2019; Iranifar et al., 2019). In this study, publicly available gene expression data of AD patients were analyzed, and the top 500 feature genes that may affect AD pathogenesis were screened out from 28,844 genes with the mRMR algorithm. This study speculated that expression of these genes may be closely related to AD pathogenesis.

Based on mRMR analysis, this study constructed an optimal AD classifier that could accurately classify AD patients and healthy individuals among SVM, RF, and KNN classifiers via IFS analysis, and then a 14-gene signature was obtained. These feature genes were then intersected with DEGs to obtain 3 core genes (HSPB3, AEBP1, RNU1G2) which may function in AD pathogenesis. Heat shock proteins (HSPs) are important molecular chaperones that prevent protein misfolding and promote the degradation of improperly folded proteins (van Noort et al., 2017). HSPs play a role in protecting multiple sclerosis, protein folding diseases, and genetic white matter diseases (van Noort et al., 2017). Although there is no direct evidence that HSPB3, a member of the HSP family, participates in AD pathogenesis, this study observed that HSPB3 was conspicuously lowly expressed and may be critical in AD (Boelens et al., 1998). Consulting to other members of the HSPs in the nervous system, HSPB3 may protect the nervous system from Aβ by degrading Aβ, whereas HSPB3 deletion may lead to AD (Calderwood and Murshid, 2017). AEBP1 plays an important role in lipid metabolism, which activates inflammatory responses through the NF-κB pathway and regulates adipogenesis in preadipocytes (Majdalawieh and Ro, 2010; Shijo et al., 2018; Gerhard et al., 2019). AEBP1 is up-regulated in AD patients, which promotes the inflammatory response around the nucleus in hippocampal pyramidal neurons, the formation of neurofibrillary tangles, and the progression of AD (Shijo et al., 2018). This study revealed that AEBP1 was up-regulated in the MTG of AD patients. This result denoted that the inflammatory stress and adipogenesis in the MTG may result in the pathogenesis of AD. RNU1G2 is a kind of small nuclear RNA molecule (snRNA) that cannot translate itself into protein, but it participates in pre-mRNA processing. So far, there has been few discussions about the mechanism of RNU1G2 and its biological functions. However, a study manifested the changes of RNU1G2 expression in the brain of AD patients (Piras et al., 2019). This study disclosed that RNU1G2 was highly expressed in the brain of AD patients and may be critical in AD pathogenesis, indicating that alternative RNA splicing is promising to disclose the pathogenesis of AD. In conclusion, whilst some research on HSPB3 and AEBP1 has presented their roles in the pathogenesis of AD, these investigations are still insufficient. It is worth exploring the role of the above three genes in the occurrence and progression of AD.

Furthermore, RWR analysis was conducted here on a DEGs-based PPI network with the above core genes as seed genes, and the core gene-associated genes screened out were then subjected to GO and KEGG enrichment analyses. The results illustrated that these core gene-associated genes were mainly related to biological processes such as synaptic vesicle cycle, transport vesicle, and protein kinase C binding, and activated in functional pathways such as MAPK signaling pathway, B cell receptor signaling pathway, and T cell receptor signaling pathway. The above results indicated that AD may be associated with neurotransmitter transmission. The decrease of neurotransmitter and activity is an essential phenotype of AD, whereas neurotransmitter supplementation is pivotal to the treatment of AD (Kandimalla and Reddy, 2017). In this study, the three core genes we identified were related to neurotransmitter transmission, suggesting that these genes may affect AD pathogenesis by modulating neurotransmitter secretion. Moreover, the results of enrichment analysis clarified that AD was associated with immune cell stress response. Inflammation is considered to be a key factor influencing the progression of AD, and microglia activation exacerbates both Aβ and tau deposition (Kinney et al., 2018). T cells function in AD and multiple sclerosis. An investigation displayed that hippocampal T cell infiltration leads to neuroinflammation and cognitive impairments (Laurent et al., 2017). Another study suggested that Aβ serves as an antigenic factor of T cells to potentiate encephalitis (Leoutsakos et al., 2018). As such, this study exhibited that the core genes we identified were related to B cell and T cell receptor signaling pathways, indicating that the three core genes may regulate immune cell activity, thereby affecting the pathogenesis of AD.

In summary, gene expression data of AD were firstly downloaded here from GEO database. Next, an AD classifier with favorable diagnostic efficacy was screened out through mRMR and IFS. The feature genes in the classifier were intersected with DEGs to obtain three core genes (HSPB3, AEBP1, RNU1G2) closely related to AD pathogenesis. Among the three core genes, HSPB3 may regulate protein folding processes and degrade misfolded proteins. AEBP1 stimulates adipogenesis in preadipocytes to induce inflammation and activates inflammatory responses to facilitate the pathogenesis of AD. Although our studies are reliable, these results are still in need of verification by molecular biological experiments. Further research could explore the biological functions of the three genes and the pathogenesis of AD to produce findings that account more for clinical therapy, thereby benefiting AD patients.

## Data Availability Statement

The datasets presented in this study can be found in online repositories. The names of the repository/repositories and accession number(s) can be found in the article/ Supplementary Material.

## Author Contributions

All authors contributed to data analysis, drafting and revising the article, gave final approval of the version to be published, and agreed to be accountable for all aspects of the work.

## Conflict of Interest

The authors declare that the research was conducted in the absence of any commercial or financial relationships that could be construed as a potential conflict of interest.

## Footnotes

1 https://www.ncbi.nlm.nih.gov/geo/

## Supplementary Material

Supplementary Table 1 Top 500 genes selected by mRMR analysis.

Supplementary Table 2 Fifty-two core gene-associated genes obtained by RWR analysis.
